# Can sPD-1 and sPD-L1 Plasma Concentrations Predict Treatment Response among Patients with Extraparenchymal Neurocysticercosis?

**DOI:** 10.3390/pathogens12091116

**Published:** 2023-09-01

**Authors:** Andrea Toledo, Gladis Fragoso, Roger Carrillo-Mezo, Matthew L. Romo, Edda Sciutto, Agnès Fleury

**Affiliations:** 1 Unidad de Neuro Inflamación, Departamento de Medicina Genómica y Toxicología Ambiental, Instituto de Investigación Biomédicas, Universidad Nacional Autónoma de México (UNAM)/Instituto Nacional de Neurología y Neurocirugía, Ciudad de México 14269, Mexico; antoro06@yahoo.com; 2 División de Investigación, Facultad de Medicina, Universidad Nacional Autónoma de México (UNAM), Ciudad de México 04510, Mexico; 3 Departamento de Inmunología, Instituto de Investigación Biomédicas, Universidad Nacional Autónoma de México (UNAM), Ciudad de México 04510, Mexico; gladis@unam.mx (G.F.); edda@unam.mx (E.S.); 4 Departamento de Neurorradiología, Instituto Nacional de Neurología y Neurocirugía, Ciudad de México 14269, Mexico; mayroger@hotmail.com; 5 Department of Epidemiology & Biostatistics, CUNY Graduate School of Public Health and Health Policy, City University of New York, New York, NY 10027, USA; matthew.l.romo@gmail.com; 6 Clínica de Neurocisticercosis, Instituto Nacional de Neurología y Neurocirugía, Ciudad de México 14269, Mexico

**Keywords:** neurocysticercosis, *Taenia solium*, treatment, biomarker, PD-1/PD-L1

## Abstract

Extraparenchymal neurocysticercosis (EP-NC) is a chronic, potentially life-threatening disease that responds poorly to initial anthelmintic drug therapy. A depressed specific reactivity of peripheral lymphocytes and an increased level of specific Tregs accompanies EP-NC. The immune checkpoint pathway PD-1 and its ligand PD-L1 downregulates effector T cells, causing specific immune suppression in chronic diseases. This study explored whether their soluble forms, sPD-1/sPD-L1, are present in plasma among patients with EP-NC and if their levels could be associated with treatment response. A total of 21 patients with vesicular EP-NC and 22 healthy controls were included. Patients received standard treatment and were followed for six months to assess treatment response by assessing changes in cyst volume determined with 3D MRI. The presence of both sPD-1 and sPD-L1 was more frequently detected among patients with EP-NC than in healthy controls and had higher concentrations. Among patients, higher pre-treatment levels of both markers were associated with a poor treatment response, and the sensitivity and specificity of the sPD-1/sPD-L1 ratio for predicting any response to treatment were high. Our results are consistent with the presence of lymphocyte exhaustion and open new research perspectives to improve the prognosis of patients with this severe disease.

## 1. Introduction

Neurocysticercosis (NC) is a disease caused by the presence of Taenia solium larvae in the central nervous system. The parasite’s life cycle takes place in rural communities, where pigs are free-ranging and fecal matter disposal is precarious. Because of this characteristic, this parasitosis is still endemic in most countries of Latin America, Asia, and sub-Saharan Africa. One of the characteristics of NC is its considerable clinical heterogeneity. Various factors contribute to this, principally the intensity of the inflammatory reaction associated with the presence of the parasite, and the location of the parasite larvae, which may be found inside or outside the parenchyma (extraparenchymal NC, EP-NC). EP-NC is the most severe form of NC [1], first because of the potential severity of symptoms and second because of the frequent poor response to anthelmintic treatment [2,3].

We previously found that EP-NC occurs with a concomitant depressed reactivity to parasitic antigens, an increased CD25(high)Foxp3+ Treg differentiation, and a higher DC expression of the regulatory molecules signaling lymphocytic activation molecule family 1 (SLAMF1) and CD205, favoring a suppressive environment [4,5]. Specific stimulation of PBMCs from these patients does not induce the production of inflammatory cytokines (IL-5, IL-6, TNF-a), whereas this is the case in healthy controls [5]. Furthermore, some studies have highlighted the role of the pro-inflammatory response in the response to specific treatment of these EP-NC patients [6].

Programmed death protein 1 (PD-1 or CD 279) is a membrane-bound receptor expressed primarily on activated T lymphoid cells that has been identified as an immune checkpoint, playing an important role in inhibiting cellular activity [7]. PD-1 is a member of the B7 family which interacts specifically with its co-stimulatory ligands, PD-Ligand 1 (PD-L1) and 2 (PD-L2), which are ubiquitously expressed and necessary for maintaining peripheral tolerance. These ligand–receptor interactions result in reduced immune cell activity and thus provide an anti-inflammatory environment that is critical for the maintenance of immunological homeostasis. It is, however, now clear that aberrant expression of PD-1 and/or its ligands is involved in different pathological processes and in the response to their treatment [8,9]. The presence of soluble forms of PD-1/PD-L1 (sPD-1 and sPD-L1) have also been reported, although their precise function is still under debate [10,11].

In this context, the main objective of this work was to evaluate the possible relationship between sPD-1/sPD-L1 and both other peripheral immune markers and treatment response among patients with EP-NC.

## 2. Materials and Methods

### 2.1. Patients and Controls

The patients included were a subgroup of those included in previous studies [3,5] and the reason why only a subgroup was included was due to the limited availability of plasma. Recruitment and general characteristics of the study population have been previously described [3,5]. Briefly, a group of 21 patients with confirmed EP-NC [12] were included prior to the administration of a standard regimen of anthelmintic treatment (albendazole) plus corticosteroids and followed for at least 6 months to evaluate their responses to treatment via 3D-MRI to estimate the parasite volumes. A group of 24 healthy controls without NC was also included for comparison. The inclusion criteria for the patients were to have a diagnosis of vesicular EP-NC based on validated diagnostic criteria [12], to accept the 15-day hospitalization period and standard medical treatment, to not have been treated with anthelmintic drugs (albendazole, praziquantel, or ivermectin) and corticosteroids in the past 6 months, to agree to attend follow-up visits at 1 and 6 months after treatment, and to agree to give blood samples before treatment and during the two follow-up visits. As controls, asymptomatic family members of patients attending INNN, as well as staff and students, were invited to participate. Exclusion criteria for all the individuals included were uncontrolled metabolic diseases, neoplastic diseases, neurological and infectious diseases (including NC), and pregnancy.

The mean age was 47.52 years (SD: 10.5) among patients and 45.63 years (SD: 9.8) among controls, with no statistically significant difference (*p* = 0.536). Males comprised 61.9% of patients and 62.5% of controls, with no statistically significant difference (*p* = 0.967). All patients had active extraparenchymal NC (EP-NC), with viable parasites localized only in the subarachnoid space for 15 (71.4%), only in the ventricles for 2 (9.5%), and in both locations for 4 (19.1%). At inclusion, their primary neurological symptom was headache (17 [81.0%]), dizziness (2 [9.5%]), and epilepsy (2 [9.5%]). For 16 patients, the diagnosis of NC had been made recently (1–3 months prior to inclusion), while for 5 patients, the diagnosis had been made 2–5 years prior to inclusion in the study. These 5 patients had already received specific treatment but did not receive treatment (anthelmintic and corticosteroid) for at least 6 months before inclusion.

The study protocol was approved by the ethical committee of Instituto Nacional de Neurología y Neurocirugía (63/14). All participants provided written informed consent for their neurological and radiological information and blood samples to be used for research purposes.

### 2.2. Plasma Samples

A 10 mL venous blood sample drawn from participants was obtained in tubes coated with ethylenediamine tetra-acetic acid (EDTA) (BD Vacutainer). The samples were kept at room temperature and processed within 2 h. The samples were centrifuged at 800 *g* for 10 min, aliquoted, and frozen at −80 °C until use.

### 2.3. Evaluation of sPD1 and SPD-L1 in Plasma

Levels of sPD-1 and sPD-L1 before and 6 months after treatment were evaluated in the 21 patients. Both soluble molecules were also measured 1 month after treatment in 12 of the 21 patients. Commercial sandwich enzyme linked immunosorbent assay (ELISA) kits were employed to quantify, in plasma, the concentrations of sPD-1 and sPD-L1 (R&D Systems, bio-techne, Minneapolis, MN 55413, USA), following the supplier’s instructions. Sandwich ELISAs were performed in 96-well, flat-bottom microtiter plates (Nunc-Immuno Plate Maxisorp). The detection limits were 78 pg/mL for both sPD-1 and sPD-L1. All analyses were run in duplicate.

### 2.4. Cytokine Titration and Peripheral Immunologic Profile

The cytokine titration and the evaluation of the specific peripheral immunologic profile were performed as described previously [5]. Briefly, the pro- and anti-inflammatory human cytokines IL-1β, IL-4, IL-5, IL-6, IL-17A, CCL5, and INF-γ (all from BioLegend, San Diego, CA, USA) were determined with commercial sandwich enzyme linked immunosorbent assay (ELISA) kits, following the supplier’s instructions. For the specific immunologic profile, the PBMC cells stimulated with *T. solium* vesicular fluid or medium alone was measured using standard phenotyping protocols provided by the manufacturers. Cellular populations of naive cells (CCR7+/CD45RA+), central memory (CCR7+/CD45RA−), effector (CCR7−/CD45RA−/CD27+/CD28+), B cells (CD19+), T Regs (CD4+/CD25+/FoxP3+), and NK (CD3−/CD16+/CD56+) were determined.

### 2.5. Statistical Analysis

We computed descriptive statistics for sPD-1 and sPD-L1 detectability, levels, and the sPD-1/sPD-L1 ratio. Pearson’s chi-square tests were used to compare the detectability of sPD-1 and sPD-L1 between participants with EP-NC and healthy controls. Mann–Whitney U tests were used to compare sPD-1 and sPD-L1 levels and ratios of these levels between participants with EP-NC and healthy controls. Among participants with EP-NC, Wilcoxon signed rank tests were used to evaluate changes in levels among the three time points (before treatment, 1 month post-treatment, and 6 months post-treatment). Kruskal–Wallis tests were used to compare sPD-1 and sPD-L1 levels and the sPD-1/sPD-L1 ratio by treatment response categories at three time points. Spearman’s rank correlation coefficients were used to evaluate the association between sPD-1 and sPD-L1 levels and the sPD-1/sPD-L1 ratio with percent reduction in cyst volume and peripheral immunologic parameters. Receiving operating characteristic (ROC) curves analysis was used to evaluate the sensitivity and specificity of the studied markers to predict response to treatment (any response, complete response) and determine the optimal cut-off value. Data were compiled in an Excel sheet and analyzed using SPSS and GraphPad prism software. A *p* < 0.05 was considered statistically significant.

## 3. Results

### 3.1. Differences in sPD-1 and sPD-L1 Presence and Concentration between Patients and Controls

sPD-1 and sPD-L1 were evaluated in the 21 patients before treatment and in the 24 controls. sPD-1 was not detected in 5 (23.8%) of the patients and sPD-L1 was not detected in 6 (28.6%) of the patients, while in the controls, both sPD-1 and sPD-L1 were not detected in 16 (66.7%) of them. Detection of sPD-1 and sPD-L1 was significantly more frequent among patients than controls (*p* = 0.007 and *p* = 0.02, respectively).

**Figure 1:**
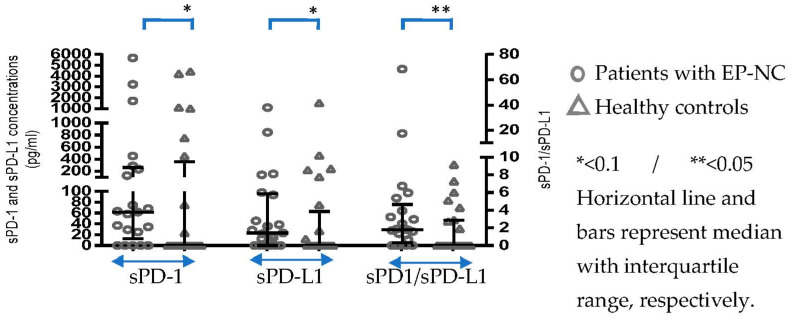
Differences in sPD-1 and sPD-L1 plasma concentrations and in the sPD-1/sPD-L1 ratio between patients with EP-NC (before treatment) and healthy controls.

Figure 1 illustrates the differences in sPD-1 and sPD-L1 concentrations between patients before treatment and healthy controls. Concentrations of both were higher in patients (sPD-1: Median: 62; IQR: 12.5–259.7/sPD-L1: Median: 23.5; IQR: 0–95.8) vs. controls (sPD-1: Median: 0; IQR: 0–356.2/sPD-L1, Median: 0; IQR: 0–93.7) and had borderline statistical significance (*p* = 0.056 and *p* = 0.06, respectively). The ratio of sPD-1/sPD-L1 was significantly higher (*p* = 0.039) in patients (median: 1.77; IQR: 0.32–4.66) vs. controls (median: 0; IQR: 0–0).

### 3.2. Correlation between Presence of sPD-1/sPD-L1 and Response to Treatment

We evaluated the correlations between levels of sPD-1 and sPD-L1 measured at the different time points and percentage of reduction of cysts volumes between pre-treatment and 6 months post-treatment periods. All the correlations were negative (showing associations between higher response to treatment and lower levels of both markers), and the only statistically significant correlation was between levels of sPD-1 and reduction of cyst volume at 1 month post treatment (r = −0.584, *p* = 0.046).

**Figure 2:**
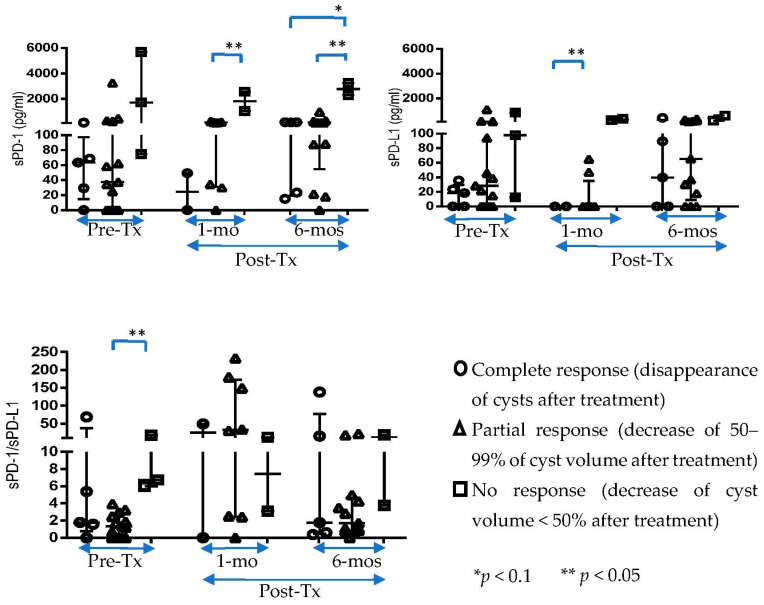
Changes in sPD-1, sPD-L1, and sPD-1/sPD-L1 over the three time points, stratified by treatment response.

We categorized patients in three groups based on treatment response [complete response (disappearance of all cysts), partial response (decrease of 50–99% of cyst volumes after treatment), and no response (decrease of <50% of cyst volumes after treatment)] and compared the levels of markers among them (Figure 2). Levels of sPD-1 and sPD-L1 were higher in non-responders than in partial or complete responders. This was significant (*p* < 0.05) compared with partial responders for both markers at 1 month post-treatment (sPD-1: no response, Median: 1802, IQR: 1042–2563; partial response: Median: 134.1, IQR: 31.08–174.3/sPD-L1: no response, Median: 277.5, IQR: 217.5–337.5; partial response: Median: 0, IQR: 0–35.25) and at 6 months post treatment for sPD-1 (no response, Median: 2769, IQR: 2293–3245/partial response, Median: 111, IQR: 54.6–176). Before treatment, the ratio of sPD-1/sPD-L1 was significantly higher (*p* = 0.0018) in non-responders (Median: 6.73, IQR: 5.97–17.46) compared with partial responders (Median: 1.36, IQR:0–2.72).

### 3.3. Correlation between Concentration of sPD-1/sPD-L1 and the Specific Peripheral Immunologic Profile

Negative correlations with a *p* < 0.1 were observed between sPD1, sPD-L1, or their ratio and specific immunological pro-inflammatory parameters (Supplementary Table S1). Pre-treatment sPD-1 and sPD-L1 (and their ratio) were negatively correlated with CCL5 and NK cells (before treatment), TNF-α, IL-1β, and %NK cells (at 1 month post treatment), and with IL-17, IL-6, CCL5, IFN-γ, proliferative index, and %NK cells (6 months post-treatment). The only positive correlation occurred between sPD-1/sPD-L1 ratio and IL-4 (an anti-inflammatory cytokine) when evaluated 1 month post-treatment. For sPD-1, sPD-L1, and their ratio at 1 month post-treatment, negative correlations were observed with the following inflammatory parameters: TNFα and IL-6 (1 and 6 months post treatment). Negative correlations were also observed with IL-4 (6 months post treatment) and positive ones with IFN-γ (before treatment and at 1 month post treatment).

### 3.4. Sensitivity and Specificity of Pretreatment sPD-1/sPD-L1 to Predict Treatment Response

**Figure 3:**
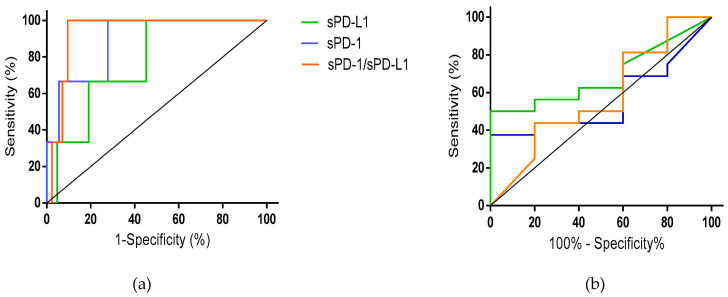
ROC curve analysis for the different pretreatment markers (sPD-1, sPD-L1, and sPD-1/sPD-L1) for the prediction of response to treatment. (**a**) Any treatment response to treatment, i.e., decrease in the volume of cysts at 6 months after treatment; (**b**) Complete disappearance of cysts at 6 months after treatment.

We dichotomized the response to treatment arbitrarily, redefining the responders as patients with any decrease in cyst volume at 6 months posttreatment. Only three patients were considered complete non-responders, and all the responder patients had a decrease in cyst volume >50%. (Figure 3a). For sPD-1, the area under the curve (AUC) was 0.89 (95% CI 0.72–1), significantly different from chance (*p* = 0.03). The best sensitivity (100%) and specificity (72.2%) were obtained using a cut-off point of 71.55 ng/mL. Regarding sPD-L1, the AUC was 0.77 (95% CI 0.55–0.98) and not statistically significant (*p* = 0.12). Using a cut-off point of 95.8 ng/mL, we obtained a sensitivity of 66.7% and a specificity of 78.6%. Regarding the ratio of sPD-1/sPD-L1, the AUC was 0.94 (95% CI 0.86–1), significantly different from chance (*p* = 0.01). With a cut-off point of 5.67 ng/mL, we can predict response to treatment with a sensitivity of 100% and a specificity of 90.48%. 

We then conducted the same analysis for predicting complete treatment response (disappearance of all cysts at 6 months after treatment). For the three markers, the area under the curve was not significantly different from chance (Figure 3b; sPD-1: AUC 0.56 (95% CI 0.30–0.82), *p* = 0.679; sPD-L1: AUC 0.69 (95% CI 0.46–0.92), *p* = 0.215; sPD-1/sPD-L1: AUC 0.57 (95% CI 0.27–0.88), *p* = 0.62).

### 3.5. Evolution sPD-1 and sPD-L1 Concentrations and sPD-1/sPD-L1 Ratio over Time by Treatment Response

When we examined levels of sPD-1 and sPD-L1 and their ratio over time, we did identify significant changes among patients overall or by treatment response (Figure 2).

## 4. Discussion

The difficulty in treating patients affected by EP-NC is one of the main problems that prevails in the clinical management of NC. Although albendazole is effective, a single cycle of treatment allows only a 30% response for EP-NC, and several cycles are generally necessary for a complete response [3]. Surgery is an option when cysts are located in the ventricular system [13], but when cysts are located in the subarachnoid cisterns, it is generally impossible to extract all the cysts and the risk of complications is high. 

In this study, we found that the presence of sPD-1 and sPD-L1 was significantly more frequent in EP-NC patients before treatment and that their concentrations were higher compared with healthy controls. Moreover, among patients, concentrations of sPD-1 and sPD-L1 was positively associated with treatment response, and their sensitivity and specificity to determine absence of response to treatment was high. 

These findings represent the first time that markers able to predict the response to anthelminthic treatment in these very serious patients are described. Our results could have relevant positive implications, helping to inform therapeutic decisions. Indeed, the presence of a high concentration of sPD-1 and/or sPDL-1 before treatment could indicate the use of a more prolongated therapeutic regimen as was previously suggested [14], or to combine albendazole with praziquantel, as it was shown to be more efficacious than anthelminthic monotherapy in parenchymal NC [15]. Also, it might be possible to reduce the doses of steroids or the duration of their administration in those patients with higher levels of sPD-1. On the other hand, the finding of a high concentration at 1 month post treatment could indicate the need to repeat the anthelminthic cycle without waiting the typical 6 months after treatment. 

We also found several negative correlations between pre- and 1 month post-treatment concentrations of sPD-1 or sPD-L1 or their ratio and components of the inflammatory reaction (cytokines and cells). This association was previously shown in other contexts [16], and although in a previous study we did not find statistically significant associations between cytokine concentrations and response to treatment [5], these results may be related to the depressed immune status of these patients with severe disease and could contribute to the chronicity of the disease. 

In several cancers, high sPD-1 and/or sPD-L1 concentrations have shown to be markers of poor prognosis [17,18] and of poor response to treatment using immune checkpoint inhibitors [19,20,21,22]. In cancers, sPD-1 functions as a blocker of PD-1 ligands and can suppress the interactions of PD-1, with PD-L1 and PD-L2 enhancing the activity of specific TCD8 cells [23]. Thus, the functional role of the soluble forms of these checkpoint markers may depend on the circumstances associated with each pathology, the magnitude of the relationship between the sPD-1/membrane-bound PD-1 and sPD-L1/membrane-bound PD-L1, and the immunoinflammatory environment. Based on these results, further studies will be needed to compare the membrane vs. the soluble respective forms. 

In different parasitic diseases, the relevance of PD-1/PD-L1 in pathogenesis has also been evaluated. In experimental cutaneous leishmaniasis caused by *L. amazonensis*, it was shown that the parasite induced PD-1 expression on lymphocytes and that treatment with anti-PD-1 and anti-PD-L1 antibodies significantly decreased parasite loads [24,25]. In severe chronic human infection with *Trypanosoma cruzi* (Chagas disease), a gradual loss of CD8+ T cells occurred, characterized by impaired cytokine production and increased inhibitory receptor expression, particularly of PD-1 and CTLA-4 [26,27]. Therefore, immune therapeutic approaches targeting PD-1/PD-L1 pathway were evaluated in an experimental model. The results were negative, as enhancement of immune response against the parasite did not occur and parasite load in different organs was not changed [28]. Regarding helminthiasis, a few studies have been conducted. In patients affected by cystic echinococcosis, sPD-L1 was significantly higher in patients than in controls [29]. In pediatric patients affected by cystic echinococcosis, a progressive significant decrease of sPD-1 and sPD-L1 occurred after successful surgery, which was not seen when relapse of the disease occurred after treatment [30]. In an experimental model of murine alveolar echinococcosis (*E. multilocularis*) infection, anti-PD-L1 administration results in increased CD4+/CD8+ effector T cells and decrease of T regs, as an improved control of the infection [31]. In cysticercosis, one study using the experimental model of *Taenia crassiceps* provided evidence that infection induces macrophage alternatively activated with strong suppressive activity involving the PD-1/PD-L1 pathway [32]. Also, in patients with NC compared to controls, higher blood levels of regulatory T cells that express higher levels of cytotoxic T lymphocyte antigen 4 (CTLA-4), lymphocyte-activation gene 3 (LAG-3), programmed death 1 (PD-1), and glucocorticoid-induced tumor necrosis factor receptor (GITR) were observed [33], which, together with the relative low specific proliferative response [5], precludes an immunosuppressive state that may favor parasite persistence.

Altogether these results allowed us to propose that lymphocyte exhaustion occurred in patients with EP-NC, very likely linked to its chronicity and to its poor response to anthelmintic treatment [3,34]. 

Our results should be considered in the context of some limitations, including the retrospective design and small sample size.

In conclusion, the results obtained herein give us hope that sPD-1/sPD-L1 may be good markers to predict the success of anthelmintic treatment for EP-NC and thus improve the management of patients with this severe disease. Further studies are needed to confirm these original findings by assessing sPD-1/sPD-L1 concentrations and PD-1 and PD-L1 expression levels in the biological fluids of a larger group of EP-NC patients.

## Data Availability

The data presented in this study are available on request from the corresponding author.

## Supplementary Materials

The following supporting information can be downloaded at: https://www.mdpi.com/article/10.3390/pathogens12091116/s1, Table S1: Correlations between pre-treatment and 1 month post-treatment levels of sPD-1, sPD-L1, and their ratio with different immunological markers.
