# Supplementary material for: Can sPD-1 and sPD-L1 Plasma Concentrations Predict Treatment Response among Patients with Extraparenchymal Neurocysticercosis?
Source: Pathogens. 2023 Sep 1;12(9):1116. doi: 10.3390/pathogens12091116 (PMC10535301; doi:10.3390/pathogens12091116)
Supplement: Supplementary file 1 [file pathogens-12-01116-s001.zip › pathogens-2509266-supplementary.pdf]

Table S1. Correlations between pre-treatment and 1-month post-treatment levels of sPD-1, sPD-L1, and their ratio with different immunological markers

	Pre-treatment			1-month post-treatment		
	**sPD-1**	**sPD-L1**	**sPD-1/sPD-L1**	**sPD-1**	**sPD-L1**	**sPD-1/sPD-L1**
**IL-1β**<br>Pre-Tx	NS	NS	NS	NS	NS	NS
1-month post-Tx	NS	r= -0.44<br>p= 0.058	NS	NS	NS	NS
6 months post-Tx	NS	NS	NS	NS	NS	NS
**IL-17A**<br>Pre-Tx	NS	NS	NS	NS	NS	NS
1-month post-Tx	NS	NS	NS	NS	NS	NS
6 months post-Tx	r= -0.50<br>p= 0.027	r= -0.67<br>p= 0.001	NS	NS	NS	NS
**CCL5**<br>Pre-Tx	NS	NS	r= -0.38<br>p= 0.09	NS	NS	NS
1-month post-Tx	NS	NS	NS	NS	NS	NS
6 months post-Tx	NS	NS	r= -0.42<br>p= 0.07	NS	NS	NS
**IL-6**<br>Pre-Tx	NS	NS	NS	NS	NS	NS
1-month post-Tx	NS	NS	NS	NS	NS	r= -0.53<br>p= 0.08
6 months post-Tx	r= -0.40<br>p= 0.079	r= -0.40<br>p= 0.075	NS	r= -0.58<br>p= 0.04	NS	NS
**TNF-α**<br>Pre-Tx	NS	NS	NS	NC	NC	NC
1-month post-Tx	r= -0.57<br>P= 0.020	r= -0.60<br>p= 0.014	NS	r= -0.61<br>p= 0.04	NS	NS
6 months post-Tx	NS	NS	NS	r= -0.59<br>p= 0.04	NS	NS
**IL-4**<br>Pre-Tx	NS	NS	NS	NS	NS	NS
1-month post-Tx	NS	NS	r= 0.44<br>p= 0.049	NS	NS	NS
6 months post-Tx	NS	NS	NS	NS	r= -0.54<br>p= 0.07	NS
**IFN-γ**<br>Pre-Tx	NS	NS	NS	NS	r= 0.68<br>p= 0.01	NS
1-month post-Tx	NS	NS	NS	r= 0.67<br>p= 0.01	r= 0.60<br>p= 0.04	NS
6 months post-Tx	NS	NS	r= -0.50<br>p= 0.03	NS	NS	NS
**% Proliferative**<br>Pre-Tx	NS	NS	NS	NS	NS	NS
1-month post-Tx	NS	NS	NS	NS	NS	NS

6 months post-Tx	r= -0.48<br>p= 0.041	r= -0.40<br>p= 0.09	r= -0.41<br>p= 0.09	NS	NS	NS
**% Naïve**<br>Pre-Tx	NS	NS	NS	NS	NS	NS
1-month post-Tx	NS	NS	r= -0.56<br>p= 0.02	NS	NS	NS
6 months post-Tx	NS	NS	NS	NS	NS	r= -0.57<br>p= 0.05
**% Central memory**<br>Pre-Tx	NS	NS	NS	NS	NS	NS
1-month post-Tx	NS	NS	NS	NS	NS	NS
6 months post-Tx	NS	NS	NS	NS	NS	NS
**% Effector memory**<br>Pre-Tx	NS	NS	NS	NS	NS	NS
1-month post-Tx	NS	NS	NS	NS	NS	NS
6 months post-Tx	NS	NS	r= -0.64<br>p= 0.01	NS	NS	NS
**% NK**<br>Pre-Tx	NS	NS	r= -0.54<br>p= 0.036	NS	NS	NS
1-month post-Tx	NS	NS	r= -0.47<br>p= 0.074	NS	NS	NS
6 months post-Tx	NS	NS	NS	NS	NS	NS
**% Tregs**<br>Pre-Tx	NS	NS	NS	NS	NS	NS
1-month post-Tx	NS	NS	NS	NS	r= -0.57<br>p= 0.07	NS
6 months post-Tx	NS	NS	NS	NS	NS	NS

Only results with a $p<0.1$ are presented. NS designates a result with a $p \geq 0.1$.

IL-5, %Bregs, and %NKT were also assessed, and all correlations were $p \geq 0.1$.
